# Shenfu injection improves isoproterenol-induced heart failure in rats by modulating co-metabolism and regulating the trimethylamine-N-oxide - inflammation axis

**DOI:** 10.3389/fphar.2024.1412300

**Published:** 2024-06-20

**Authors:** Lin Li, Jiahao Ye, Zhenyu Zhao, Siyuan Hu, Hao Liang, Ji Ouyang, Zhixi Hu

**Affiliations:** ^1^ Provincial Key Laboratory of TCM Diagnostics, Hunan University of Chinese Medicine, Changsha, Hunan, China; ^2^ The Domestic First-class Discipline Construction Project of Chinese Medicine, Hunan University of Chinese Medicine, Changsha, Hunan, China; ^3^ Post-Graduate School, Hunan University of Chinese Medicine, Changsha, Hunan, China

**Keywords:** gut microbiota, 16S rDNA, metabolomics, inflammation, Shenfu injection

## Abstract

Heart failure (HF) is a chronic condition that progressively worsens and continues to be a major financial burden and public health concern. The “gut-heart” axis provides an innovative perspective and therapeutic strategy for preventing and treating heart failure. Shenfu injection (SFI) is a Traditional Chinese Medicine-based treatment demonstrating potential as a therapeutic strategy for heart failure. However, the precise therapeutic mechanisms of SFI in heart failure are not completely characterized. In this study, HF models were established utilizing subcutaneous multipoint injection of isoproterenol (ISO) at a dosage of 5 mg kg^−1^·d^−1^ for 7 days. Serum levels of inflammatory biomarkers were quantified using protein microarrays. Rat feces were analyzed using untargeted metabolomics research and 16S rRNA sequencing. The link between gut microbiota and metabolites was examined using a MetOrigin and Spearman correlation analysis. Our results show that Shenfu injection effectively enhances cardiac function in rats with ISO-induced heart failure by potentially modulating pro-/anti-inflammatory imbalance and reducing serum and urine Trimethylamine-N-oxide (TMAO) levels. Moreover, SFI significantly increases the abundance of *Bacteroidota* at the phylum level, thereby improving disrupted gut microbiota composition. Additionally, SFI supplementation enriches specific genera known for their capacity to produce short-chain fatty acids. SFI was found to be associated with three key metabolic pathways, as revealed by fecal metabonomics analysis, including the pentose phosphate pathway, pyrimidine metabolism, and purine metabolism. Metabolite tracing analysis revealed that Taurine and hypotaurine metabolism was found to be specific to the microbial community. The biosynthesis of Pyrimidine metabolism, Purine metabolism, beta-alanine metabolism, Naphthalene degradation, Pantothenate, and CoA biosynthesis were identified as co-metabolic pathways between microbes and host. The Spearman correlation analysis was also significantly correlated to differentially expressed metabolites regulated by SFI and the gut microbiota. These results suggest that SFI improves ISO-induced heart failure by modulating co-metabolism and regulating the TMAO-inflammation axis.

## Graphical Abstract

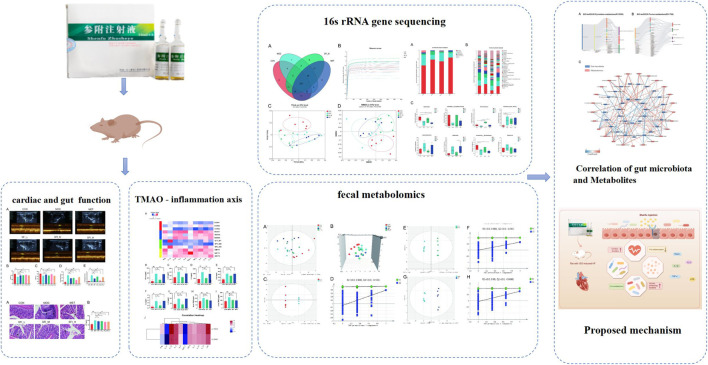

## 1 Introduction

Heart failure (HF) is a complex and potentially fatal condition caused by a variety of cardiovascular disorders. It is marked by high costs, substantial morbidity and mortality, and therefore presents a significant worldwide health concern (Groenewegen et al., 2020). Over 64 million people worldwide are affected by chronic heart failure (CHF), and despite ongoing advancements in diagnosis, treatment, and management, re-admission and mortality rates have not decreased considerably (Savarese et al., 2023). Therefore, identifying potential therapeutic targets for heart failure is crucial for preventing disease progression.

The expanding focus on the potential role of the gastrointestinal system in the development of HF has sparked increasing interest (Tang et al., 2019). The gut-heart axis, which describes the interaction between the gut and the heart, presents a unique viewpoint and potential therapeutic avenue for managing heart failure (Madan and Mehra, 2020; Bui et al., 2023). Tang et al. pioneered the “gut hypothesis of heart failure” which postulated that reduced cardiac output in heart failure could compromise intestinal perfusion, resulting in mucosal ischemia and subsequent damage to the intestinal mucosa. Intestinal barrier dysfunction can cause increased permeability, leading to malnutrition, bacterial translocation, and higher levels of endotoxins in the bloodstream (Tang et al., 2019). This can trigger inflammation linked to heart failure, with factors like epithelial dysfunction, gut barrier compromise, microbiota imbalance, and abnormal gut metabolites playing a role in the development and worsening of the condition (Tang et al., 2019; Cui et al., 2023). Increased systemic inflammation is reported to be linked to gut dysbiosis, especially in HF patients (Madan and Mehra, 2020).

Trimethylamine-N-oxide (TMAO) is a metabolite derived from the gut microbiota, originating from phosphatidylcholine, choline, betaine, and L-carnitine. These nutrients are abundant in seafood, dairy products, egg yolks, muscle meat, and organ meats. The gut microbiota’s trimethylamine (TMA) lyase hydrolyzes these nutrients to form the TMAO precursor TMA, which is further oxidized by hepatic flavin monooxygenase to form TMAO (Zhang et al., 2021). It has been suggested that dietary choline and TMAO may contribute to cardiovascular disease (CVD) (Canyelles et al., 2023). The increase in blood TMAO levels has been linked to increased inflammatory genes and cytokines, resulting in increased oxidative stress (Saaoud et al., 2023). The pathophysiology of numerous inflammatory conditions is considerably affected by the activation of inflammatory pathways and the production of inflammatory cytokines by TMAO. The development of heart failure has been linked, in part, to inflammation, thus, alleviating inflammation is crucial for enhancing the clinical manifestations and prognosis of HF. Consequently, targeting the TMAO-inflammation axis could be a unique therapeutic strategy for treating heart failure.

Based on recent research, Traditional Chinese Medicine (TCM) can prevent the development of cardiovascular diseases by regulating the gut microbiota and reducing inflammation (Huang et al., 2020). TCM is a valuable tool for improving cardiac function, mitigating medical conditions, and improving the overall wellbeing of patients. In treating post-acute myocardial infarction heart failure, recent pharmacological evaluations—such as those conducted by Wu et al. (2022)—have demonstrated the reliability and effectiveness of SFI, highlighting its potential to improve heart function and reduce associated symptoms (Wu et al., 2022). Researchers have shown that TCM is effective in modulating the dysbiosis of gut microbiota, stimulating the proliferation of beneficial microorganisms, suppressing the proliferation of detrimental microorganisms, harmonizing the abundance of commensal and pathogenic bacteria, and sustaining a favorable gut milieu (Huang et al., 2020; Jia et al., 2020). Shenfu injection (SFI) is one of the representative prescriptions of the warming Yang method. It originates from the classic formula Shenfu Decoction and continues to be applied extensively in the treatment of heart failure with remarkable curative effects (Luo et al., 2021). Currently, SFI is a formulation created with *Panax ginseng* C.A. Mey [Araliaceae; Ginseng radix et rhizoma rubra] and *Aconitum carmichaelii* Debx [Ranunculaceae; Aconiti lateralis radix praeparata] (Chen et al., 2017) utilizing cutting-edge technologies. Previous pharmacological experiments have confirmed that Shenfu Injection can enhance myocardial contractility, increase cardiac output, and inhibit cardiomyocyte apoptosis caused by myocardial ischemia/reperfusion injury, among other cardiovascular effects (Wu H. et al., 2019; Wang et al., 2021). In addition, a study by Zhu et al. demonstrated that this intervention can mitigate myocardial injury through the prevention of mitochondrial apoptosis and facilitating vasodilation by augmenting eNOS activity via the PI3K/Akt signaling pathway (Zhu et al., 2020). Nevertheless, the precise underlying mechanism remains incompletely understood. Therefore, this research endeavors to delve into the mechanisms underlying the enhancement of cardiac function in chronic heart failure through the administration of SFI.

## 2 Material and method

### 2.1 Shenfu injection

Shenfu injection (batch number:221111AK05) was purchased from Huarun Sanjiu Pharmaceutical Co., Ltd. (Ya’an, Sichuan, China). This injection is a solution extracted from *Panax ginseng* C.A. Mey and *Aconitum carmichaelii* Debeaux, as described in Table 1. The two Chinese herbs—P*anax ginseng* C.A. Mey and *Aconitum carmichaelii* Debeaux—in crude form were soaked and concentrated into solutions of 1 mg/mL and 2 mg/mL, respectively, and then mixed to form the SFl injection (Huang et al., 2024). All voucher specimens are deposited in the herbarium center of Huarun Sanjiu Pharmaceutical Co., Ltd. The plant was identified was conducted by a botanist at Huarun Sanjiu Pharmaceutical Co., Ltd.

**TABLE 1:** Information of raw herbs in SFl.

Latin name	Plant part	English name	Chinese name	Ratio (%)	Origin of place	Month of harvest
*Panax ginseng* C.A Mey	Root	Red Ginseng Root	Hongshen	33.3	Jilin, China	September
*Aconitum carmichaelii* Debeaux	Tuber	Aconite	Fuzi	66.6	Sichuan, China	August

### 2.2 Reagents and chemicals

Metoprolol tartrate was bought from AstraZeneca Pharma (NO2302069) (Jiangsu, China). Isoproterenol (C_11_H_17_NO_3_▪HCL) was purchased from Aladdin (Shanghai Aladdin Biochemical Technology Co., Ltd., Shanghai, China). Protein chip detection: A protein chip assay kit (QAR-INF-1) was purchased from Raybiotech, Inc. (Norcross, GA, United States of America). The TMAO kit was provided by Xiamen Luncanshuo Biotech Co., Ltd. The NT-proBNP and LPS kit was provided by Wuhan Huamei Biological Engineering Co., Ltd.

### 2.3 Animals and ethics statement

This study received approval from the Ethical Committee of Hunan University of Chinese Medicine (Hunan, China) under Approval Number LL202309230002. Thirty-two male Sprague-Dawley (SD) rats, aged 6 weeks that weighed 200–220 g were procured from Hunan SJA Laboratory Animal Co. Ltd. (Hunan, China) and maintained under standard husbandry conditions.

### 2.4 Quality control of the shenfu injection

Ultra-High Performance Liquid Chromatography Quadrupole-Orbitrap Mass Spectrometry (UHPLC-QE-MS) was performed to control the quality of the Shenfu injection. The analysis was performed using a Vanquish UHPLC system (Thermo Fisher) with a Waters UPLC BEH C18 column (Xiao et al., 2020). Samples (5 μL) were eluted at 0.5 mL/min with a mobile phase of 0.1% formic acid in water (A) and acetonitrile (B). A Q Exactive Focus mass spectrometer with Xcalibur software was used for MS and MS/MS data acquisition (IDA mode, m/z range: 100–1,500). The top three ions per cycle were selected for MS/MS.

### 2.5 Establishment of animal model and drug administration

**FIGURE 1:**
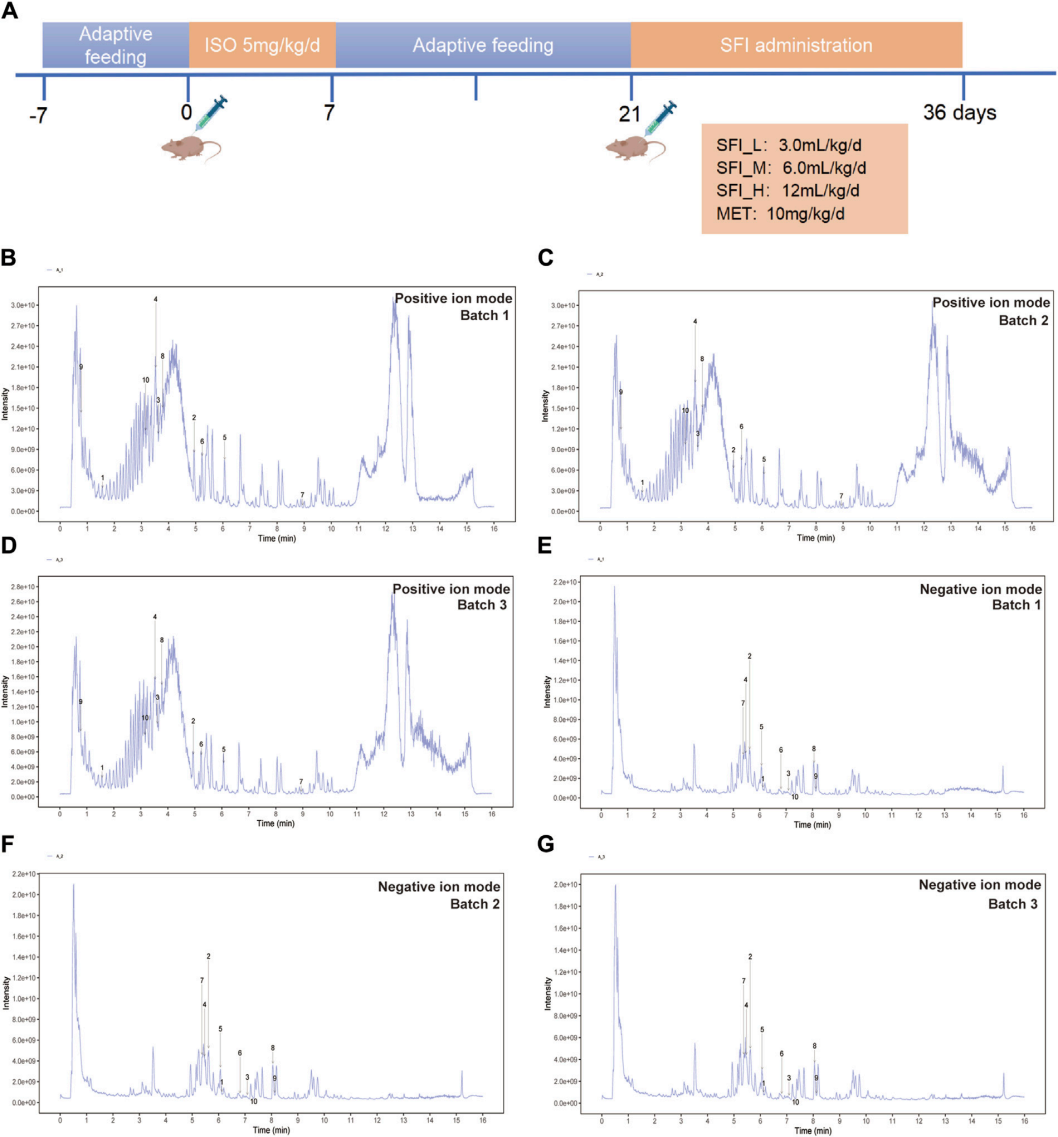
**(A)** Schematic illustration of the animal experimental design. **(B)** The UHPLC-QE-MS chromatogram illustration of three batches of SFI samples detected in positive ion mode **(B–D)** and negative ion mode **(E–G)**.

The animals in the study were divided into six groups at random (n = 8), namely, the control group (CON group), model group (MOD group), positive drug group (metoprolol, the beta-adrenergic antagonist, MET group, 10 mg/kg) (Lujan and DiCarlo, 2020), the Shenfu injection low dose group (3.0 mL/kg, SFI_L group), the Shenfu injection middle dose group (6.0 mL/kg, SFI_M group), and the Shenfu injection high dose group (12.0 mL/kg, SFI_H group). According to prior research, rats were administered subcutaneous multipoint injections of isoproterenol at a dosage of 5 mg·kg-1·day-1 for 7 days to induce the heart failure model (Huang et al., 2022). After adaptive feeding continued for 2 weeks, the CON and MOD groups were administered distilled water by gavage and had normal saline injected intraperitoneally, while the SFI group was intraperitoneally injected with SFI and administered purified water by gavage, the MET group was injected with metoprolol intragastrically and intraperitoneally with sterile water for 15 days (Figure 1A).

### 2.6 Sampling and bioassays

At the end of the experimental period, the rats were euthanized using urethane anesthesia (1.0 g/kg, i.p.). The blood samples were obtained from the abdominal aorta and allowed to clot at room temperature (25°C ± 2°C). Subsequently, centrifugation was performed to obtain serum. Myocardial and colonic tissues were fixed in a 4% paraformaldehyde solution for 48 h. Hematoxylin and eosin (H&E) staining was performed on tissue sections fixed in paraffin.

The scoring of cardiac pathological changes was based on the criteria established in the study by Rezkalla et al. (1990). Three high-power fields were observed in each slice to calculate the percentage of inflammatory infiltration and necrosis area compared to the total area. A scoring system was established as follows: 0 points for no lesion, one point for a lesion area of less than 25%, two points for a lesion area of 25%–49%, three points for a lesion area of 50%–75%, and four points for a lesion area greater than 75% (Niu et al., 2017; Xiao et al., 2019). After being extracted from the colon, fecal samples were kept in liquid nitrogen at −80°C. Six rats were randomly chosen from each group for 16S rRNA sequencing and microbiome analysis.

### 2.7 Echocardiography

For the calculation of the Left Ventricular Ejection Fraction (LVEF)and Left Ventricular Fractional Shortening (LVFS), the computer software (VINNO6, Suzhou VINNO Technology Co., Ltd., Suzhou, China) was used (Li et al., 2023).

### 2.8 UHPLC-OE-MS fecal metabolic profiling

Ultra-High Performance Liquid Chromatography-Ion Trap-Orbitrap Mass Spectrometry (UHPLC-OE-MS) was peformed using HPLC system connected to a Waters ACQUITY UPLC BEH Amide column coupled to Orbitrap Exploris 120 mass spectrometer (Orbitrap MS, Thermo). Conditions for UHPLC-MS analysis can be found in Supplementary Material (Supplementary Material 1). A Quality control (QC) mixture was created by combining extracts from all samples to assess variability. Supplementary Figure S1A illustrates the remarkable stability and reproducibility of the instruments utilized in this investigation. The distinct clustering in the QC samples demonstrates consistent and reliable performance.

Results were processed as specified in previous publications (Li et al., 2021). In this study, 15,533 in two (positive and negative) ion modes were detected, and 8,888 metabolites were found after relative standard deviation de-nosing. Metabolite identification was carried out using the R program and BiotreeDB (V3.0) (Chen et al., 2023). Biotree company in Shanghai, China, provided support for the LC-MS detection.

The Metabolomics dataset was analyzed using SIMCA14.1 software from Umetrics, Sweden. Peak numbers, sample identifiers, and normalized peak areas were imported for analysis. Principle Component Analysis (PCA) and Orthogonal Projections to Latent Structures Discriminate Analysis (OPLS-DA) were used, followed by a seven-fold cross-validation to determine *R* ^2^ and Q^2^ values. Metabolites with Variable Importance in the Projection (VIP) > 1.0 and *p* < 0.05 were considered significantly changed. Identified markers were then analyzed using MetaboAnalyst to pinpoint the metabolic pathways they influenced. The origin and function analysis of metabolites was performed using MetOrigin (Liu et al., 2023). This methodology aligns with the approach described in a previous publication (Zhao et al., 2023).

### 2.9 16S rRNA gene sequencing

Tiangen Fecal Genomic DNA was used to obtain total genomic DNA following the protocols provided by the manufacturer (Liang et al., 2022). After total DNA extraction from the samples, specific primers containing barcodes were created using the complete primer sequences. The following steps included PCR amplification, purification, quantification, and normalization to generate a sequencing library. The libraries underwent quality control before being sequenced using the PacBio Sequel II system. The output data was converted to CCS files using SMRT Link analysis software, which were then used to identify samples based on their barcode sequences and converted to FASTQ format data. UCHIME v4.2 software was used to detect and eliminate chimera sequences. The resulting effective CCS sequences were used for further analysis (Edgar, 2013). Based on the species annotation information of 16S rDNA sequences in the Silva database (https://www.arb-silva.de/), the operational taxonomic units (OTUs) were classified into various taxonomic levels, including phylum, class, order, family, and genus (Ma et al., 2022). Alpha diversity was assessed using Chao, ACE, Simpson, and Shannon indices. Chao and ACE indices estimate species richness, while Simpson and Shannon indices gauge diversity. The Biotree company in Shanghai, China, provided support for the 16S rRNA gene sequencing.

### 2.10 Protein chip detection

The protein chip assay kit QAR-INF-1 was utilized to measure the levels of inflammatory factors in four distinct groups. The assay was carried out following the manufacturer’s protocol.

### 2.11 Statistical analysis

Results were presented as mean ± SD. Statistical analyses were performed using IBM SPSS Statistics 25. The Kolmogorov-Smirnov test was employed to evaluate the normal distribution of the data. Parametric tests were applied to normally distributed data, whereas non-parametric tests were used for data that did not exhibit normal distribution. One-way ANOVA or Dunnett’s T3 test was used based on variance homogeneity. Differences with *p* < 0.01 were substantially significant, while *p* < 0.05 was significant. Correlations were calculated using Spearman’s rank correlation (presented as Spearman rho). Prism GraphPad software was used for figure creation.

## 3 Results

### 3.1 Qualitative detection of SFI under LC-MS conditions

UHPLC-QE-MS analysis was employed for the quality control assessment of SFI. Figure 1B presents representative base peak intensity chromatograms of three different batches of SFI samples. These chromatograms demonstrated effective separation and detection of major SFI components in both positive and negative ion modes, using optimized chromatographic and MS conditions (Figures 1B–G). The chromatograms clearly indicated stable and consistent composition across the three SFI batches. The core compounds were selected for Total ion chromatogram (TIC) icon peak labeling, the details on the compounds are illustrated in Table 2.

**TABLE 2:** The core compounds of SFI.

No	Name	MZ	RT	Formula	Type	MS2
1	(20S,23E)-3beta,12beta,20,25-tetrahydroxydammarane-23-ene 20-O-beta-D-glucopyranoside|ginsenoside Rh13	653.43	367.382	C_36_H_62_O_10_	NEG	653.427309; 668.887099; 336.878879; 537.416933; 581.417049
2	Ginsenoside F1	683.44	337.19	C_36_H_62_O_9_	NEG	683.442099; 637.424548; 92.675844; 101.024143; 475.379881
3	Chikusetsu saponin IVa	793.44	425.282	C_42_H_66_O_14_	NEG	793.432508; 71.013628; 59.013837; 101.023884; 113.023864
4	Ginsenoside Ro	955.49	328.119	C_48_H_76_O_19_	NEG	955.487604; 955.515614; 793.434039; 569.389402; 75.008669
5	Ginsenoside Re	945.54	364.179	C_48_H_82_O_18_	NEG	945.538413; 945.48327; 71.013666; 101.024208; 89.024148
6	6-Gingerol	293.18	408.432	C_17_H_26_O_4_	NEG	221.152945; 236.105298; 220.145786; 293.175077; 71.013682
7	Ginsenoside Rg2	783.49	322.2315	C_42_H_72_O_13_	NEG	783.480972; 783.50177; 475.380023; 92.678382; 59.013959
8	Ginsenoside Rg3	783.48	483.441	C_42_H_72_O_13_	NEG	783.480964; 783.501762; 101.024211; 71.013629; 113.023869
9	Ginsenoside Rg3 (R-FORM)	829.49	487.319	C_42_H_72_O_13_	NEG	783.481909; 783.502707; 101.024255; 71.013675; 113.024865
10	Ginsenoside-Rg1	845.49	439.588	C_42_H_72_O_14_	NEG	799.484899; 799.506337; 637.427691; 101.024146; 92.675001
1	Bullatine B	438.28	94.0406	C_24_H_39_NO_6_	POS	438.282997; 420.277593; 439.154571; 388.245091; 406.256222
2	Ginsenoside Rf	823.48	296.459	C_42_H_72_O_14_	POS	823.475297; 823.520123; 365.102344; 91.497063; 245.062527
3	Ginsenoside RG1	823.48	217.128	C_42_H_72_O_14_	POS	823.475313; 823.520139; 643.41145; 203.051766; 91.497901
4	Benzoylaconine	604.31	211.611	C_32_H_45_NO_10_	POS	604.317144; 105.03308; 109.100833; 95.084826; 123.11597
5	Ginsenoside Rd	969.54	364.299	C_48_H_82_O_18_	POS	969.533842; 789.468488; 789.510567; 969.476581; 970.537505
6	Ginsenoside Rb1	1,091.60	314.367	C_54_H_92_O_23_	POS	85.028256; 145.049096; 325.11008; 127.038605; 163.060486
7	14-Deoxy-11,12-didehydroandrographolide	315.20	536.445	C_20_H_28_O_4_	POS	133.100608; 315.195615; 189.090703; 157.10117; 105.070083
8	Benzoylhypaconine	574.30	226.736	C_31_H_43_NO_9_	POS	574.302648; 542.27759; 105.033157; 63.810472; 140.107385
9	Aconine	500.28	46.2104	C_25_H_41_NO_9_	POS	500.284; 450.24443; 55.586708; 468.255394; 418.218269
10	Benzoylmesaconine	590.30	189.104	C_31_H_43_NO1_0_	POS	590.293131; 105.033194; 540.259757; 65.588159; 558.264448

RT, retention time; NEG, negative ion mode; POS, positive ion mode; MS, mass spectrum.

### 3.2 Pharmacodynamic evaluation

#### 3.2.1 SFI improved cardiac function in rats with ISO-induced HF

**FIGURE 2:**
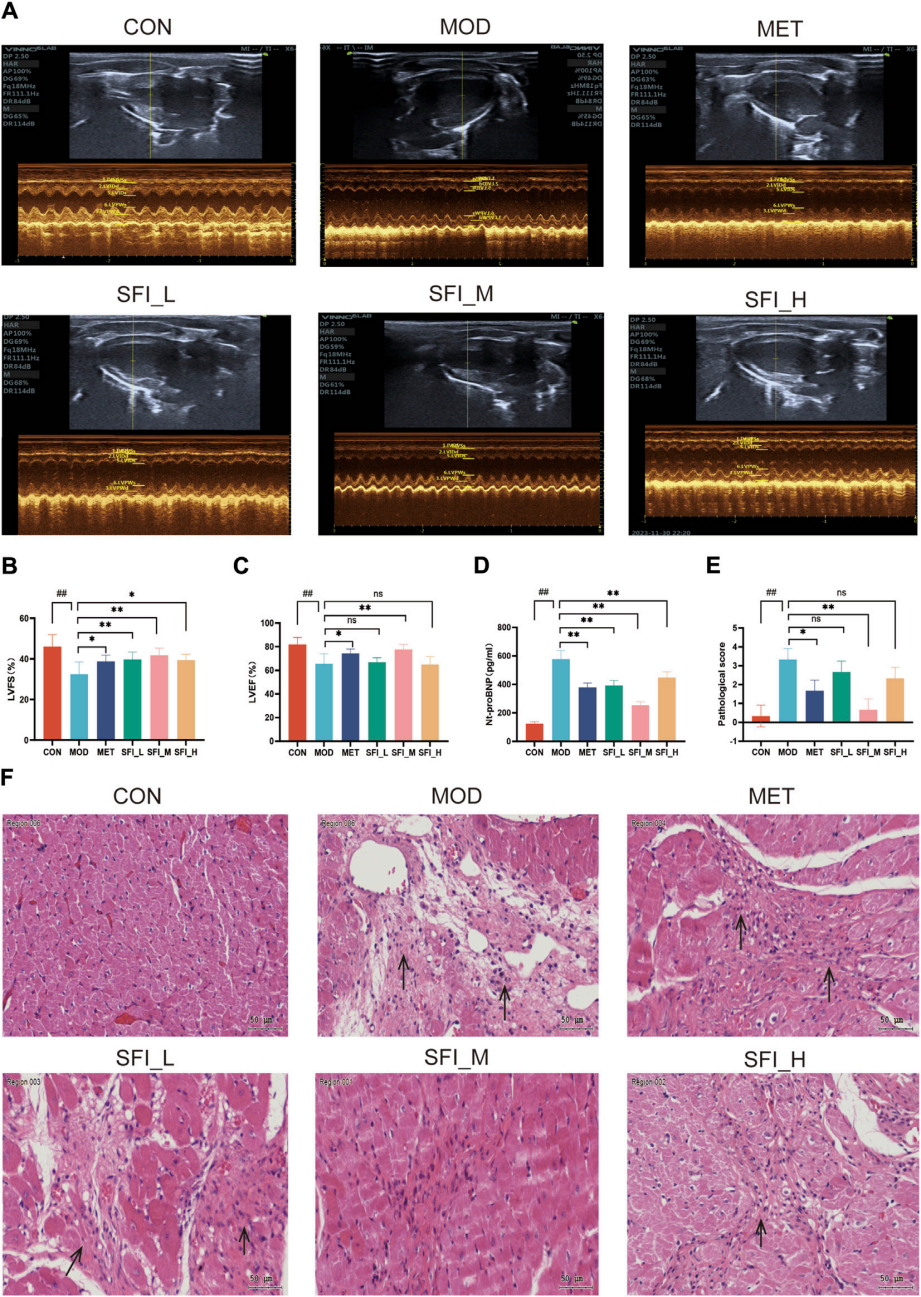
**(A)** Mode echocardiography illustration for the six groups. **(B)** Left ventricular fractional shortening. **(C)** Left ventricular ejection fraction. **(D)** Serum level of NT-proBNP, as measured by ELISA. **(E)** Pathological score of H&E staining (n = 3). **(F)** Representative H&E staining images of myocardial tissue (×200). Notes: Data were reported as mean ± SD, n = 8. ^##^ *p* < 0.01, compared with the CON group; ^**^ *p* < 0.01, compared with the MOD group.

The Representative echocardiographic images are shown in Figure 2A. As shown in Figures 2B, C, the LVEF and LVFS levels in the MOD group were significantly decreased compared to the CON group, but the LVFS levels in the SFI_L group, SFI_M group, SFI_H group, and MET group were increased compared with the MOD group (Figure 2B). However, only the SFI_M and MET groups had markedly increased LVEF levels (Figure 2C). As shown in Figure 2D, there was a notable rise in the NT-proBNP levels in the MOD group. However, treatment with SFI and MET led to a significant reduction in NT-proBNP levels in the rats, with the most pronounced effect observed in the SFI_M group.

The H&E staining in the MOD group showed myocardial dissolution and inflammation infiltrating the tissue, while myocardial structures were vastly improved after SFI and MET treatment, and the most significant effect was observed in the SFI_M group (Figures 2E, F). The improvement observed after SFI treatment was not in a dose-dependent manner.

#### 3.2.2 SFI attenuates gut function in rats with ISO-induced HF

**FIGURE 3:**
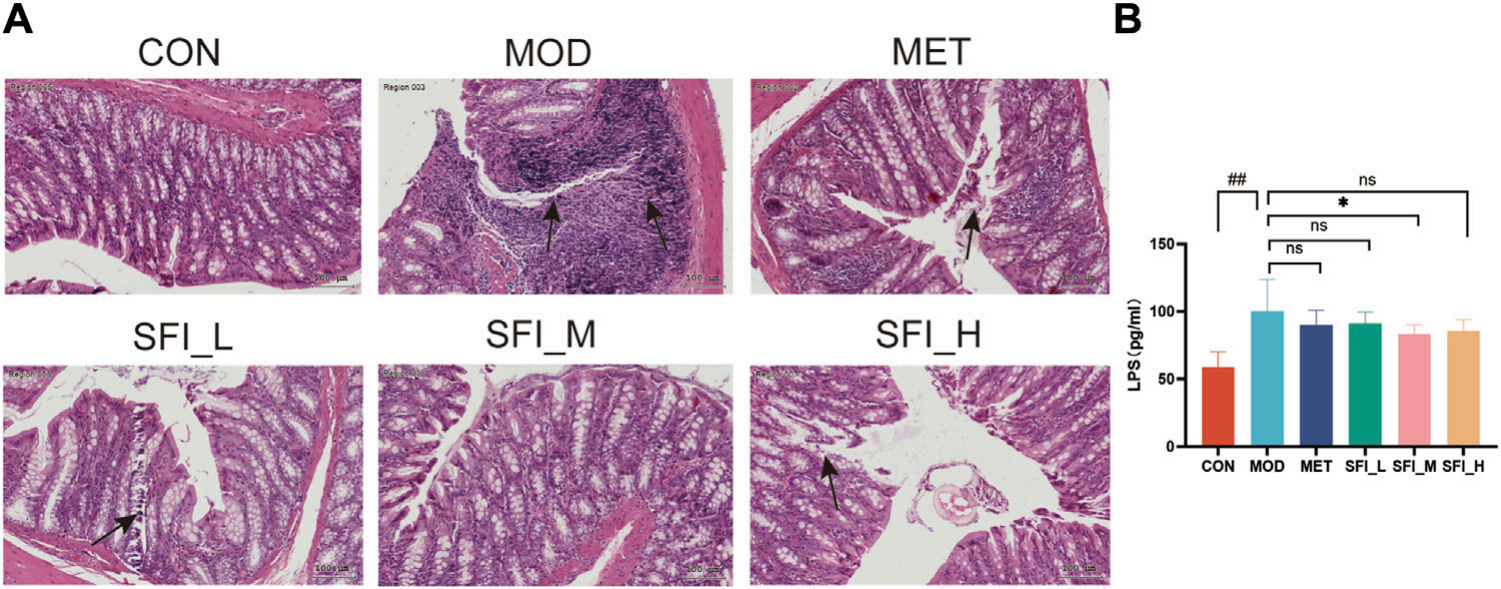
**(A)** Representative H&E staining images of colon tissue (×200). **(B)** Serum level of LPS, as measured by ELISA. Notes: Data were reported as mean ± SD, n = 8. ^##^ *p* < 0.01, compared with the CON group; ^**^ *p* < 0.01, compared with the MOD group.

The H&E-stained colonic segment of the MOD group displayed compromised intestinal mucosa integrity, inflammatory cell infiltration into the colon tissue, and lymphoid hyperplasia (Figure 3A), indicating potential damage to the intestinal barrier (leaky gut) in the MOD group. Following SFI and MET administration, the colon tissue structures were primarily improved, and the SFI_M treatment attenuated gut function better than SFI_L and SFI_H treatment. As shown in Figure 3B, the serum Lipopolysaccharide (LPS) level in the MOD group was considerably higher than in the CON group, and following SFI_M administration, there was a reduction in the level of LPS.

These findings indicate that SFI potentially preserves both cardiac and gut function in a manner that is not strictly dose-dependent, with the most significant impact observed at intermediate doses. Consequently, a dose of 6 mL/kg was selected for subsequent experiments, including cytokine microarray analysis, 16S rRNA sequencing, and metabolomics studies.

#### 3.2.3 SFI improves inflammation and TMAO levels in rats with ISO-induced HF

**FIGURE 4:**
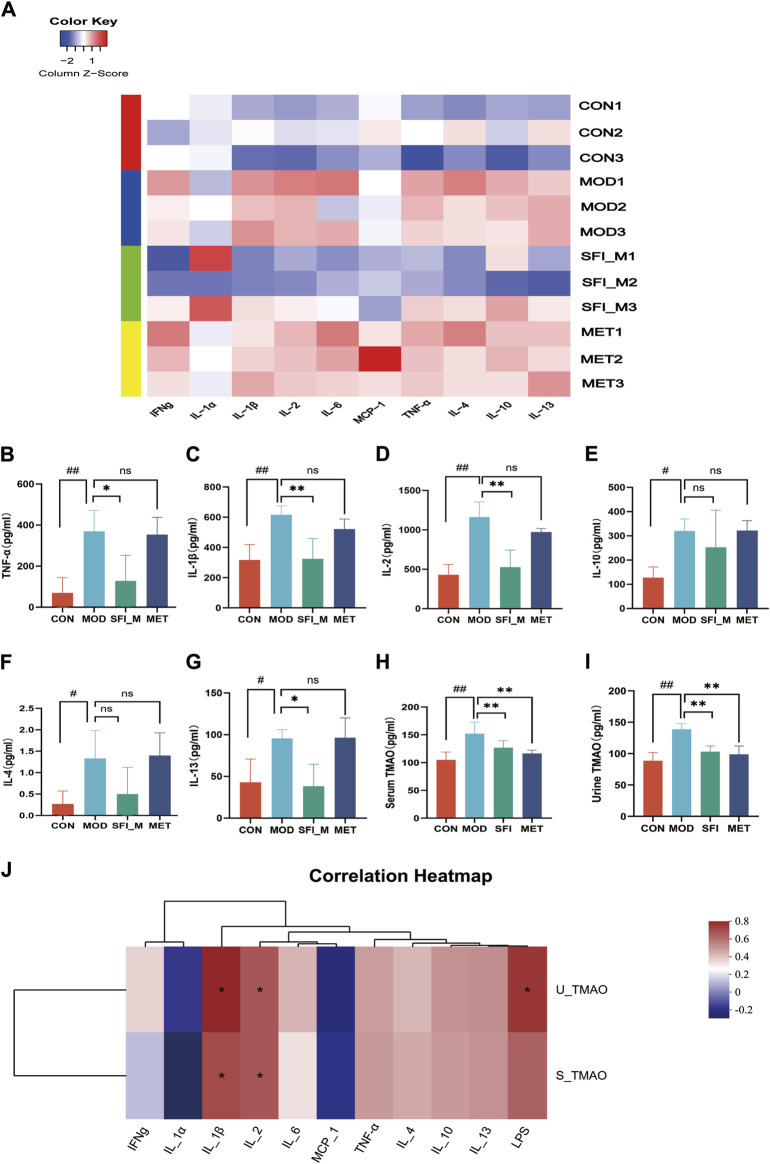
**(A)** The heatmap of inflammatory cytokines (n = 3); Cytokine microarray result of **(B)** TNF-α, **(C)** IL-1β, **(D)** IL-2, **(E)** IL-10, **(F)** IL-4 and **(G)** IL-13. **(H)** Serum level of TMAO, as measured by ELISA. **(I)** Urine level of TMAO, as measured by ELISA. **(J)** Spearman correlations between inflammatory cytokines and TMAOs.

Cytokine microarray was employed to examine the anti-inflammatory influence of SFI on rats with ISO-induced HF (Figure 4A). Figures 4B–D illustrate that the three highly expressed pro-inflammatory cytokines (TNF-α, IL-1β, and IL-2), in the serum of HF rats were significantly downregulated in the presence of SFI. However, in the HF rat model (Figures 4E–G), the anti-inflammatory cytokines IL-4, IL-10, and IL-13 were elevated, reflecting the pro-/anti-inflammation imbalance. It has been shown that the levels of TMAO were related to inflammation (Wang Q. et al., 2022). As illustrated in Figures 4H, I, TMAO levels in serum and urine in HF were substantially elevated but decreased after treatment with SFI and MET.

#### 3.2.4 Correlation analysis between TMAO and inflammation

Red denotes a positive Spearman correlation, while blue denotes a negative Spearman correlation. As shown in Figure 4J positive correlation was observed between inflammatory factors IL-1β and IL-2 and serum TMAO and urine TMAO, as well as between urine TMAO and LPS.

### 3.3 16s rRNA sequencing results

#### 3.3.1 OTU analysis

**FIGURE 5:**
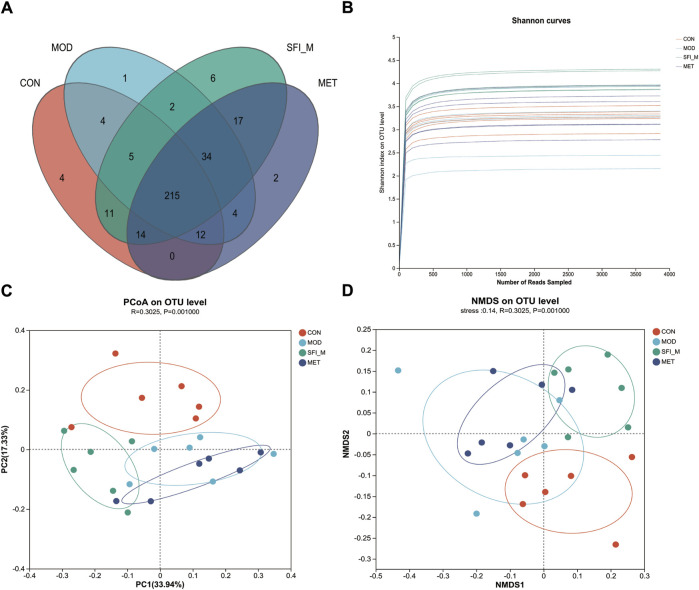
**(A)** Venn diagrams of the four groups. **(B)** The Shannon curve of the four groups. **(C)** PCoA analysis of the four groups; **(D)** NMDS analysis of the four groups (n = 6).

A 97% similarity threshold was used to identify 3,870 operational taxonomic units (representing gamma diversity). The CON, MOD, SFI_M, and MET groups had 888, 845, 1,188, and 949 OTUs, respectively. This revealed that the number of operational taxonomic units (OTUs) in HF rats was decreased, however, the number of OTUs became elevated following SFI processing (Figure 5A).

#### 3.3.2 Alpha and beta diversity analysis

The Shannon curve was generated based on OTU numbers to assess the quality of gut microbiota sequencing (Figure 5B). The results indicated a consistent and minimal fluctuation in the curve, suggesting an adequate amount of sequencing data. Alpha diversity analysis was utilized to estimate the microbial diversity within individual samples. The Chao, Shannon, Simpson, and ACE indices in Supplementary Figure S1B analysis showed that the CON and MOD groups differed from one another. Following SFI administration, there was a trend toward recovery in microbial richness, although this trend was not statistically significant. Nevertheless, weighted unifrac PCoA of beta diversity indicated a distinct divergence of the four groups. The four groups are distinctly segregated, as evidenced by ANOSIM analysis (ANOSIM: R = 0.3, *p* = 0.001). As shown in Figure 5C, the CON and MOD groups could be separated, and the SFI_M group was close to the CON group, revealing that SFI_M had a therapeutic influence. The NMDS analysis corroborated the PCoA results (model stress = 0.14 < 0.2) (Figure 5D).

#### 3.3.3 Composition of gut microbiota and their differential analysis

**FIGURE 6:**
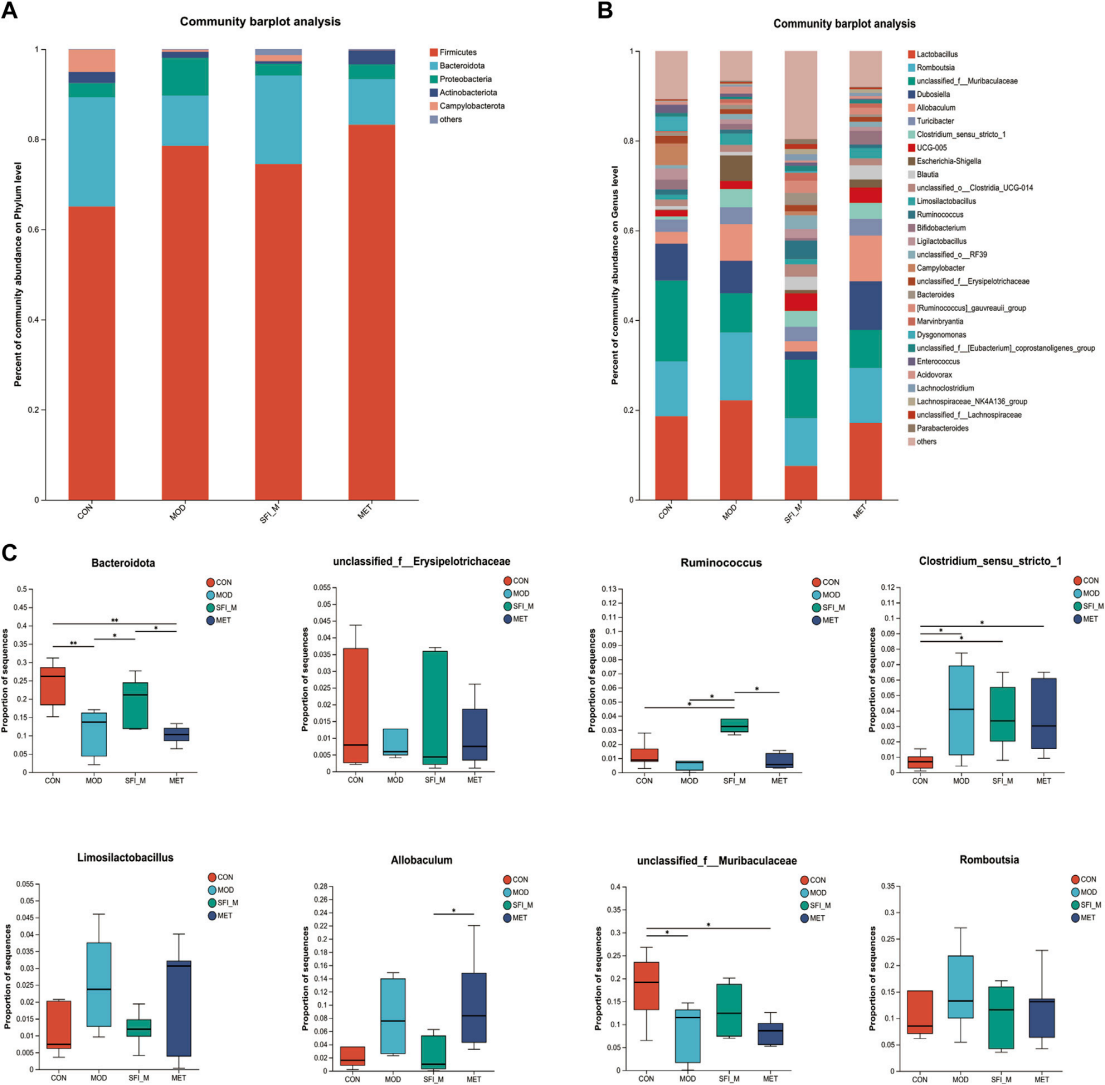
**(A)** The histogram of species distribution at the phylum and **(B)** genus levels in four groups; **(C)** Differential analysis of gut microbiota at phylum and genus level (n = 6).

The barplot in Figure 6A illustrates gut microbiota composition at the phylum and genus levels. *Firmicutes*, *Bacteroidota*, *Proteobacteria*, *Actinobacteria*, and *Campylobacterota* were the most abundant bacteria discovered at the phylum level. *Firmicutes* emerged as the predominant phylum, with relative abundances of 65.1%, 78.5%, 74.5%, and 83.2% in the CON, MOD, SFI_M, and MET groups. Furthermore, compared to the CON group, the MOD group showed a considerable decrease in bacteria with relative abundances of 24.3% and 11.2%, respectively. Additionally, treatment with SFI greatly enriched *Bacteroidota* (19.6% vs. 11.2%) compared with the MOD group.

At the genus level, there were 29 genera with an abundance of over 1% (Figure 6B). *Ruminococcus, unclassified_f_Muribaculaceae*, and *unclassified_f_* Erysipelotrichaceae were less abundant in the MOD group than in the CON group. In contrast, in the MOD group, *Romboutsia*, *Clostridium_sensu_stricto_1*, *Allobaculum*, and *Limosilactobacillus* were more productive when compared to the CON group. The levels of the microorganisms mentioned above may recover to varied degrees following SFI therapy (Figure 6C).

**FIGURE 7:**
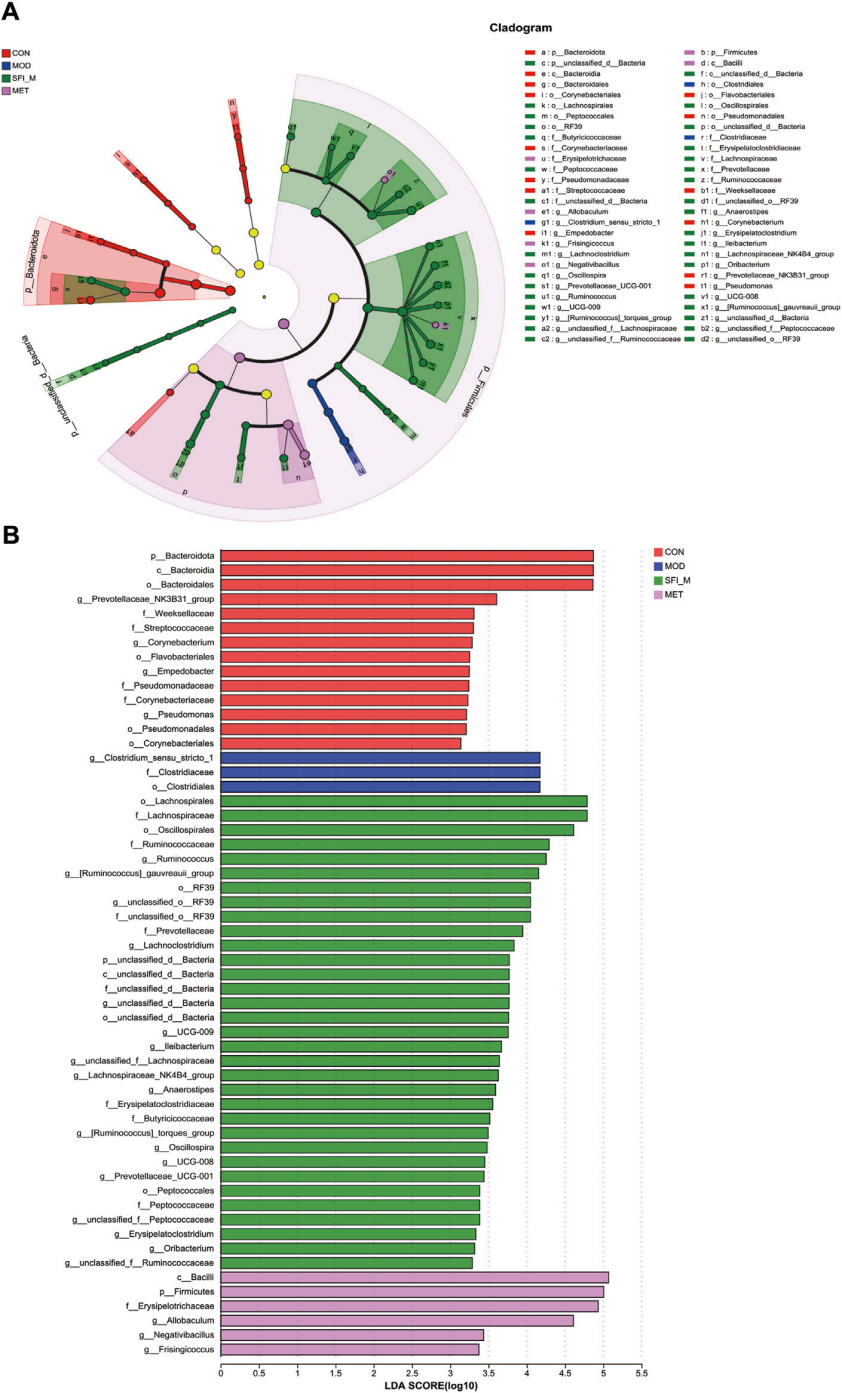
**(A)** Cladogram illustrating the phylogenetic distribution of microbiota correlated with the CON, MOD, SFI_M, and MET groups. **(B)** The variations in the abundance of microbiota among the CON, MOD, SFI_M, and MET groups (n = 6).

To ascertain the specific bacteria linked to SFI, we used linear discriminant analysis (LDA) and effect size (LEfSe) to determine the predominant taxon (Figure 7A). Our analysis revealed 56 OTUs as key discriminants, with *Bacteroidota* showing significant overrepresentation (LDA scores >4.5) in the CON group. The microbiota of Prevotellaceae_NK3B31_group was identified to be the most prevalent in the CON group as well (LDA scores >3.6) (Figure 7B). After additional LDA analysis, 15 OTUs were enriched in the CON group, 3 OTUs were enhanced in the MOD group, 24 OTUs were enriched in the SFI_M group, and 6 OTUs in the MET group. From our findings, it is evident that the microbiota in the gut of the rats can be improved through SFI intervention.

### 3.4 Study of the fecal metabolomics

#### 3.4.1 Metabolic profile analysis

**FIGURE 8:**
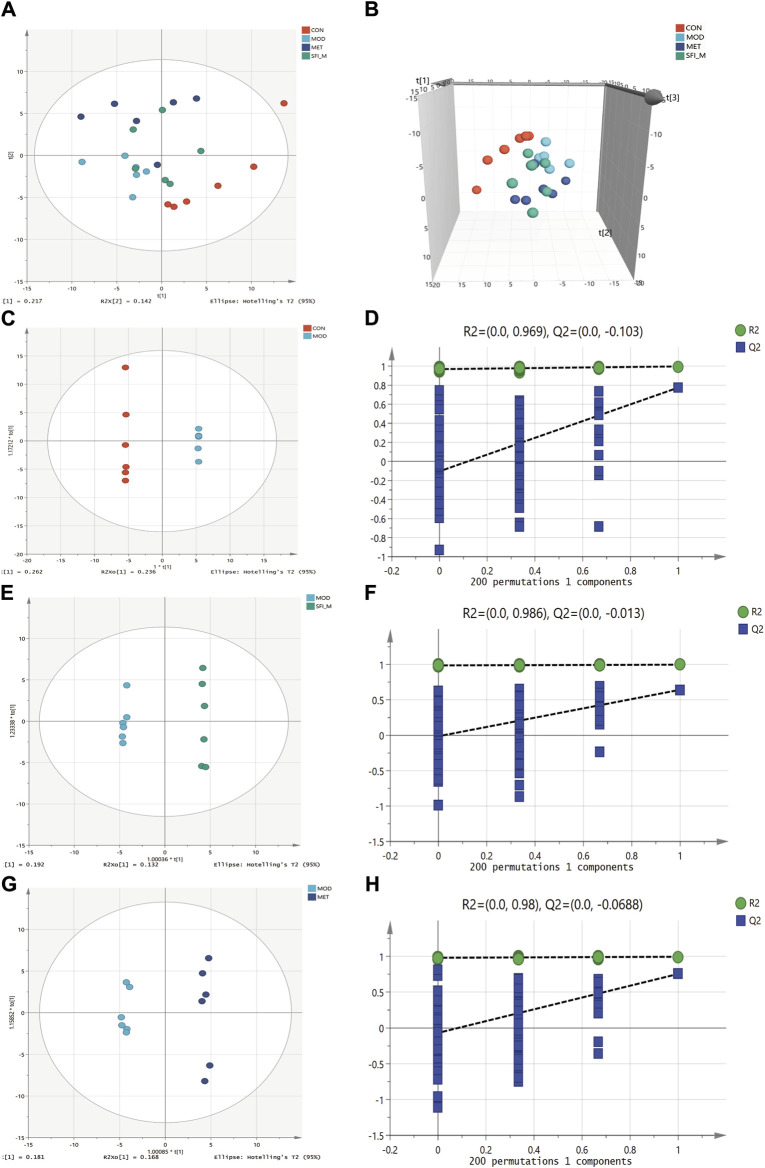
PCA score plots among four groups (R^2^X = 0.54): **(A)** 2D scatter and **(B)** 3D scatter. **(C)** OPLS-DA score plots of CON and MOD group (R^2^X = 0.753, R^2^Y = 1, Q^2^ = 0.893). **(D)** The permutation test (n = 200) for the OPLS-DA model of CON and MOD group. **(E)** OPLS-DA score plots of MOD and SFI_M group (R^2^X = 0.413, R^2^Y = 0.997, Q^2^ = 0.643). **(F)** The permutation test (n = 200) for the OPLS-DA model of MOD and SFI_M group. **(G)** OPLS-DA score plots of MOD and MET group (R^2^X = 0.486, R^2^Y = 0.995, Q^2^ = 0.755). **(H)** The permutation test (n = 200) for the OPLS-DA model of MOD and MET group (n = 6).

The metabolic changes in the four experimental groups were visualized in the current study using PCA. Figures 8A, B illustrate that the CON and MOD groups can be separated, and the SFI_M groups were scattered between them, indicating that the drug intervention regulated the metabolic disturbances induced by HF.

#### 3.4.2 Differential metabolites analysis

OPLS-DA analysis shows that the four groups were separated (Figures 8C, E, G). The OPLS-DA model was also statistically validated through 200 permutation tests, as illustrated in Figures 8D, F, H. The model was reliable. Forty-eight metabolites with VIP >1.0 and *p* < 0.05 were chosen as the metabolites linked to the pathological alteration of HF. All the metabolites showed a callback trend, and 28 metabolites were significantly changed (Table 3).

**TABLE 3:** Differential metabolites of four groups.

No	RT(s)	Var ID	Formula	KEGG	Ion form	MZ	Trend		
							M/C	S/M	M/M
1	66.8	2,4-Dimethylphenol	C_8_H_10_O	C14582	NEG	121.066	↓^##^	↑^**^	↑
2	66.8	3-(2-Hydroxyphenyl)propanoic acid	C_9_H_10_O_3_	C01198	NEG	165.056	↓^##^	↑^**^	↑
3	66.8	3-(3-Hydroxyphenyl)propanoic acid	C_9_H_10_O_3_	C11457	NEG	165.056	↓^##^	↑^**^	↑
4	242.1	Edetic acid edetic acid	C_10_H_16_N_2_O_8_	C00284	NEG	291.084	↓^##^	↑^*^	↑^*^
5	59.7	Indolelactic acid	C_11_H_11_NO_3_	C02043	NEG	204.067	↓^##^	↑^*^	↑^*^
6	249.8	2-methylcitrate	C_7_H_10_O_7_	C02225	NEG	187.025	↓^##^	↑^*^	↑
7	132.2	Caffeic acid	C_9_H_8_O_4_	C01197	NEG	179.039	↓^##^	↑^*^	↓
8	20.5	PC(15:0/P-16:0)	C_39_H_78_NO_7_P	C00157	POS	704.566	↑^##^	↓	↓
9	120	Sedoheptulose	C_7_H_14_O_7_	C02076	NEG	209.067	↓^##^	↑^**^	↑
10	111.8	D-Glucosaminic acid	C_6_H_13_NO_6_	C03752	POS	196.083	↑^##^	↓^*^	↑^*^
11	182.8	2-Hydroxyhexanedioic acid	C_6_H_10_O_5_	C02360	NEG	143.035	↓^##^	↑^*^	↑
12	146.9	Pseudo uridine	C_9_H_1_2N_2_O_6_	C02067	NEG	243.063	↑^##^	↓	↑
13	159.1	Glycocholic acid	C_26_H_43_NO_6_	C01921	NEG	464.302	↓^##^	↑	↑
14	182.8	D-Ribose	C_5_H_10_O_5_	C00121	NEG	149.046	↓^##^	↑^**^	↑
15	28.5	Gentisic acid	C_7_H_6_O_4_	C00628	NEG	153.020	↓^##^	↑^**^	↑
16	68.1	2-Hydroxyethanesulfonic acid	C_2_H_6_O_4_S	C05123	NEG	124.992	↓^#^	↑^*^	↓
17	22.6	4-Methyl-5-thiazoleethanol	C_6_H_9_NOS	C04294	POS	144.048	↓^#^	↑^**^	↑^*^
18	20.4	Arachidonic acid (AA)	C_20_H_32_O_2_	C00219	NEG	303.234	↓^##^	↑	↑
19	168.4	Urobilin	C_33_H_42_N_4_O_6_	C05794	POS	591.3187	↓^#^	↑^*^	↑
20	185.5	Tyrosine	C_9_H_11_NO_3_	C00082	POS	182.081	↓^##^	↑	↑
21	135.6	Glycochenodeoxycholic acid	C_26_H_43_NO_5_	C05466	NEG	448.307	↓^##^	↑	↑
22	21	Pyrocatechol	C_6_H_6_O_2_	C00090	NEG	109.030	↓^#^	↑	↑
23	139.9	Cholic acid	C_24_H_40_O_5_	C00695	NEG	407.282	↓^#^	↑	↑^*^
24	182.8	Fructose	C_6_H_12_O_6_	C02336	NEG	179.057	↓^##^	↑	↓
25	240.1	Nicotinamide riboside (NR)	C_11_H_15_N_2_O_5_	C03150	POS	255.097	↓^##^	↑	↓
26	163.1	Phenylalanine	C_9_H_11_NO_2_	C00079	POS	166.086	↓^#^	↑	↑
27	19.2	Phenacetin	C_10_H_13_NO_2_	C07591	POS	180.102	↓^##^	↑^**^	↑
28	294.6	S-Adenosylmethionine	C_15_H_22_N_6_O_5_S	C00019	POS	399.145	↓^#^	↑	↓
29	229.9	5-Aminopentanoic acid	C5H11NO2	C00431	POS	118.086	↓^#^	↑^**^	↓
30	134.3	Nicotinate	C6H5NO2	C00253	NEG	122.0250	↑^#^	↓	↑^*^
31	177.6	1-Aminopropan-2-ol	C3H9NO	C05771	POS	76.076	↓^#^	↑^**^	↓
32	127.4	Xanthine	C5H4N4O2	C00385	NEG	151.026	↑^##^	↓	↑^*^
33	87.5	Hypoxanthine	C_5_H_4_N_4_O	C00262	POS	137.046	↑^##^	↓^*^	↓
34	141	Mevalonic acid	C_6_H_12_O_4_	C00418	NEG	147.067	↓^#^	↑	↓
35	257.5	Histidine	C_6_H_9_N_3_O_2_	C00135	POS	156.077	↓^#^	↑	↓
36	76.7	1-Methylguanine	C_6_H_7_N_5_O	C04152	POS	166.072	↑^##^	↓^*^	↑
37	186.8	Methylimidazoleacetic acid	C_6_H_8_N_2_O_2_	C05828	NEG	139.052	↓^##^	↑	↑^*^
38	93.3	3-Hydroxyisovaleric acid	C_5_H_10_O_3_	C20827	NEG	117.056	↓^#^	↑	↓
39	190.3	Proline	C_5_H_9_NO_2_	C16435	POS	116.070	↓^#^	↑	↑
40	215.5	4-Guanidinobutyric acid	C_5_H_11_N_3_O_2_	C01035	POS	146.092	↓^#^	↑^**^	↑
41	26.4	Euscaphic acid	C_30_H_48_O_5_	C17890	POS	489.358	↑^#^	↓^**^	↓^*^
42	223.6	4-Aminobutyric acid (GABA)	C_4_H_9_NO_2_	C00334	POS	104.071	↓^##^	↑	↓
43	35.8	Uracil	C_4_H_4_N_2_O_2_	C00106	NEG	111.020	↑^#^	↓^*^	↓
44	23.4	L-tyrosine-methyl-ester	C_10_H_13_NO_3_	C03404	POS	196.097	↓^#^	↑^*^	↑
45	235.4	Chloroquine	C_18_H_26_C_l_N_3_	C07625	POS	320.185	↑^#^	↓^**^	↓
46	101.4	Deoxyinosine	C_10_H_12_N_4_O_4_	C05512	POS	253.093	↑^#^	↓^*^	↑
47	31.9	Thymine	C_5_H_6_N_2_O_2_	C00178	NEG	125.036	↑^#^	↓^**^	↑

RT, retention time; NEG, negative ion mode; POS, positive ion mode; MS, mass spectrum.

^#^ *p* < 0.05. ^##^ *p* < 0.01 compared with the CON group; *p < 0.05, **p < 0.01 compared with the MOD group. M/C: MOD group compared with the CON group. S/M: SFI_M group compared with the MOD group. M/M: MET group compared with the SFI_M group.

#### 3.4.3 Key metabolic pathway analysis for different metabolites

**FIGURE 9:**
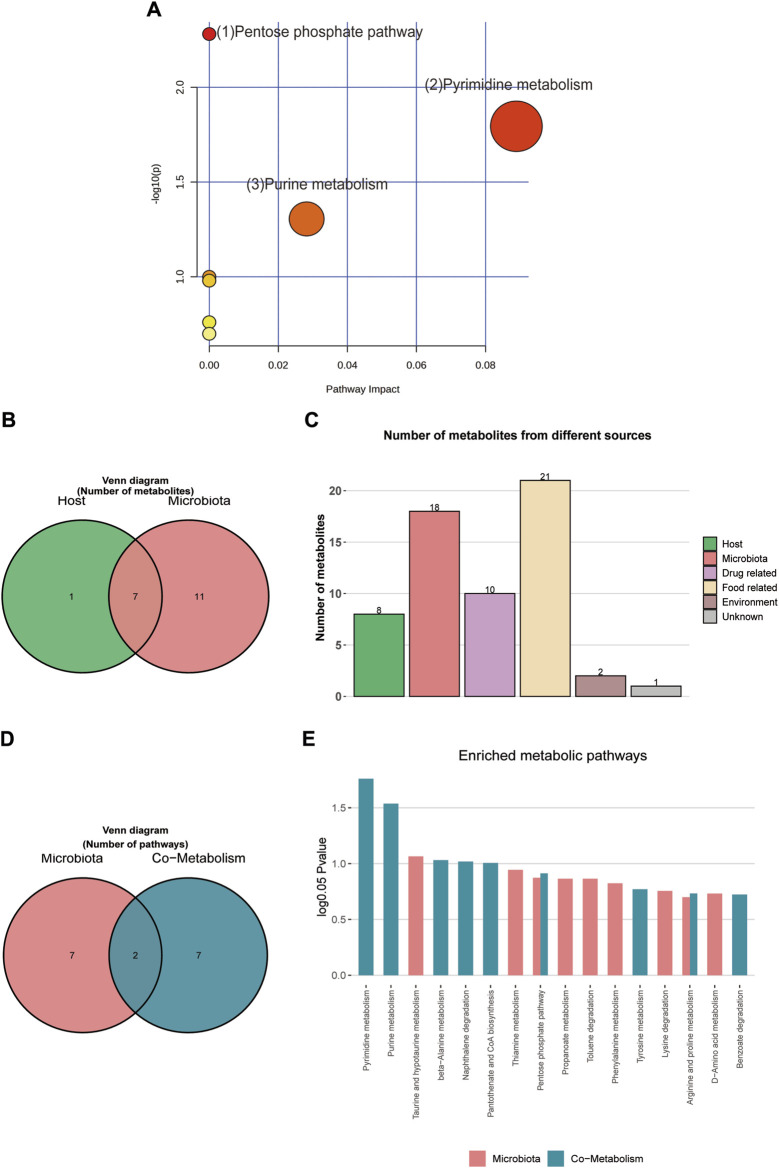
**(A)** MetPA analysis of differential metabolites pathway. **(B)** Venn diagram of differential metabolites. **(C)** Histogram of differential metabolites. **(D)** Venn diagram of enrichment analysis of differential metabolites. **(E)** Histogram of enrichment analysis of differential metabolites.

For pathway enrichment analysis, the 28 differential metabolites were imported into MetaboAnalyst 6.0 to investigate further the mechanisms underlying the SFI effects on HF. Figure 9A presents the three metabolic pathways that were influenced (*p* < 0.05): (1) Pentose phosphate pathway; (2) Pyrimidine metabolism; and (3) Purine metabolism. Supplementary Table S1 contains details on all the impacted pathways.

#### 3.4.4 MetOrigin tracing analysis of different metabolites

**FIGURE 10:**
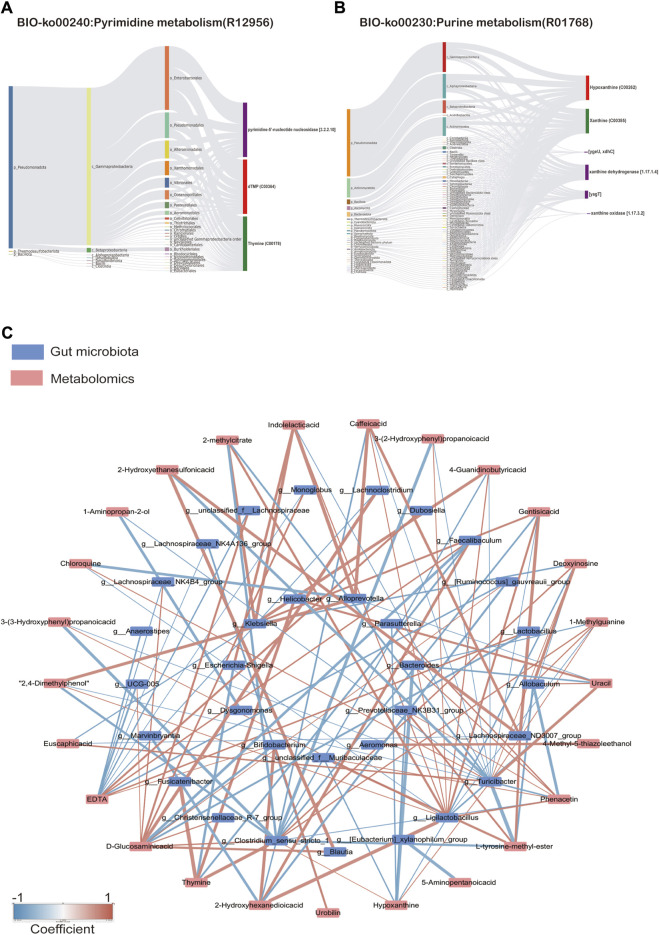
**(A)** Sankdy diagram of metorigin analysis of Pyimidine metabolism and **(B)** Purine metabolism, **(C)** Network diagram of correlation analysis (Spearman’s R-value >0.5, *p* < 0.05).

Metabolite tracing analysis identified 28 differential metabolites associated with the Shenfu injection: seven bacterial-host co-metabolites, 11 bacterial metabolites, and one host-specific metabolite (including 10 drug-related, 21 food-related, two environment-related, and one unknown) (Figures 9B, C). Metabolite pathway enrichment analysis (MPEA) showed that 7, 2, and seven relevant metabolic pathways matched with host, bacterial, and co-metabolic pathway databases, respectively (Figures 9D, E). Based on origin-based functional analysis, Taurine and hypotaurine metabolism was found to be specific to the microbial community. The biosynthesis of Pyrimidine metabolism, Purine metabolism, beta-alanine metabolism, Naphthalene degradation, Pantothenate, and CoA biosynthesis were identified as co-metabolic pathways between microbes and hosts. To better illustrate the co-metabolic relationships between microbiota and hosts, a Bio-Sankey network based on MetOrigin analysis further visualized the statistical correlations and biological relationships between microbiota and metabolites (Figures 10A, B). Supplementary Tables S2, S3 contains details of microbiota and hosts.

#### 3.4.5 Correlation analysis between metabolomics and 16S rRNA sequencing results

As shown in Figure 10C, correlation analysis revealed a close relationship between intestinal microflora and different metabolites (Supplementary Table S4). A correlation was considered significant only when the absolute value of Spearman’s correlation coefficient r was greater than 0.5 (Figure 10). When the total value of *R* was higher than 0.6, the results were considered to show a strong linear correlation between the two variables. For example, *Lactobacillus* was strongly correlated with D-Glucosaminic acid and positively correlated with EDTA. *Ligilactobacillus* was substantially inversely correlated to Hypoxanthine, Deoxyinosine, Chloroquine, Uracil, and 1-methylguanine, but positively correlated to 2-methyl citrate, Phenacetin, Caffeic acid, Gentisic acid, Indolelactic acid, 2,4-Dimethylphenol, 3-(2-Hydroxyphenyl)propanoic acid, 3-(3-Hydroxyphenyl)propanoic acid, and 4-Guanidinobutyric acid. *Clostridium*_sensu_stricto_1 was substantially inversely correlated to Hypoxanthine,1-Methylguanine, D-Glucosaminic acid, but positively correlated to 2-methylcitrate, L-tyrosine-methyl-ester, Phenacetin,2-Hydroxyethanesulfonic acid, Caffeic acid, Gentisic acid, 2,4-Dimethylphenol, 3-(2-Hydroxyphenyl)propanoic acid, 3-(3-Hydroxyphenyl) propanoic acid, and 4-Guanidinobutyric acid. These results suggest that the SFI could alter the microbe-metabolic axis.

## 4 Discussion

**FIGURE 11:**
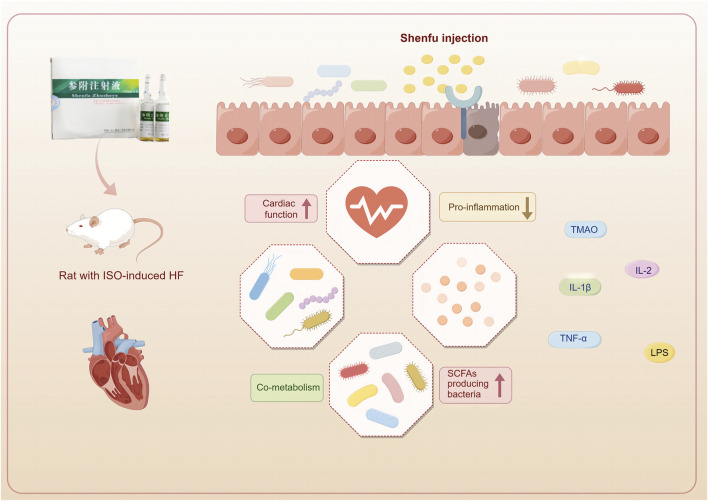
Shenfu injection improves cardiac function by modulating co-metabolism and the TMAO-inflammation axis. (This figure was drawn using Figdraw).

The prevalence of heart failure is on the rise globally due to the aging population (McDonagh et al., 2021). Emerging research indicates that this trend is significantly influenced by the gut-heart axis (Madan and Mehra, 2020). The symbiotic cooperation between the gut microbiota and the host is vital to maintaining overall health. The intricate nature of TCM poses challenges in elucidating its therapeutic mechanisms; however, using multi-omics strategies presents novel opportunities for a comprehensive understanding of the therapeutic mechanism. In this investigation, 16S rRNA sequencing and non-targeted metabolomics were employed to examine the underlying pathways of SFI treatment. The most important findings from this study are as follows (Figure 11): (1) SFI effectively enhanced cardiac function in rats with ISO-induced heart failure, (2) SFI effectively modulated inflammatory imbalance and reduced serum and urine TMAO levels, (3) Treatment with SFI altered the gut microbiota and the composition of intestinal metabolites, enriched SCFA-producing bacteria such as *Ruminococcus* and *Erysipelotrichaceae* at the genus level and influenced the metabolites particularly those associated with the pentose phosphate pathway, pyrimidine metabolism, and purine metabolism, and (4) A close relationship was established between intestinal microflora and different metabolites, indicating that the SFI could alter the microbe-metabolic co-metabolism axis.

SFI has been extensively utilized in China for more than 3 decades in the treatment of cardiovascular diseases. Its primary constituents consist of ginsenosides and aconitum alkaloids. The content of ginsenosides is 676–742 μg/mL, and the content of aconite alkaloids is 3–7 μg/mL (Song et al., 2015). Aconite has certain toxicity, but the combination of aconite and ginseng exhibits a “detoxifying and enhancing efficacy” effect (Xu et al., 2024). Ginsenosides can promote the metabolism of toxic components like aconitine, prolonging the elimination half-life of aconitine, benzoylmesaconine, and hypaconitine, thereby significantly increasing the exposure of effective components in the body. An example of ginseng’s detoxification mechanisms towards aconitine involves ginsenoside Rg1’s regulation of ion channels pathways (Xu et al., 2022). This regulation accelerates aconitine metabolism and enhances the absorption of benzoylaconine (Xu et al., 2020).To explore the effects and toxicity of the Shenfu Injection at varying concentrations on the organism, we administered three different concentrations of Shenfu Injection. Our investigation entailed assessing both cardiac function, through techniques like echocardiography, Nt-proBNP level measurements, and histological analyses of cardiac tissues using H&E staining, as well as gut function, involving examination of intestinal tissues via H&E staining and measurement of LPS levels. However, dose dependency was not obvious in this study. The moderate dose (6 mL/kg) of the Shenfu Injection exhibited the most significant improvement in cardiac and gut function.

The metabolite TMAO, produced in a process dependent on gut microbiota, is linked to the pathogenic causes of HF, and it may operate as a predictive marker for identifying patients at risk of disease advancement (Zhang et al., 2021). Results from a systematic review indicate that TMAO is related to HF severity, brain natriuretic peptide (BNP), estimated glomerular filtration rate (eGFR), and adverse outcomes such as hospitalizations and fatalities (Anderson et al., 2022). Research conducted in different population groups indicates that higher plasma TMAO levels are correlated positively to inflammation, impaired endothelial function, type 2 diabetes, central obesity, and high blood pressure (Thomas and Fernandez, 2021). Research has indicated that TMAO further induces vascular inflammation by activating the NLRP3 inflammasome (Chen et al., 2017). Overexpressing SIRT3 alleviated the activation of the endothelial NLRP3 inflammasome induced by TMAO (Wu J. et al., 2019). In our study, we observed increased levels of TMAO in the serum and urine of rats with heart failure. However, significant downregulation of TMAO levels in both serum and urine, along with pro-inflammatory cytokines TNF-α, IL-2, and IL-1β, was observed in SFI-treated HF rats, aligning with previous research findings (Yang et al., 2019). Spearman correlation analysis demonstrated positive associations between inflammatory factors IL-1β and IL-2 with serum and urine TMAO, highlighting the pivotal role of the TMAO-inflammation axis targeted by SFI.

The 16S rRNA sequencing results demonstrated a reduction in the quantity of OTUs and Alpha diversity changes (including Chao, Shannon, Simpson, and ACE), indicating the dysfunction of gut microbiota in HF rats. A previous study has reported similar findings (Li et al., 2021). At the phylum level, *Firmicutes* in the intestine primarily specialize in the hydrolysis of carbohydrates and proteins. *Bacteroides*, on the other hand, predominantly target steroids, polysaccharides, and bile acids, which aid in the absorption of polysaccharides and contribute to protein synthesis within the body. In our research, when compared to the CON group, the *Firmicutes* were found to increase and the *Bacteroidota* reduced substantially in the MOD group. Shenfu Injection significantly increased the levels of *Bacteroidota*. A research study discovered that infants with heart failure had significantly altered intestinal microbiota compared to those without heart disease, with an increased number of *Firmicutes* at the phylum level (Zhang et al., 2023). At the genus level, *Ruminococcus,* *Erysipelotrichaceae, and unclassified_f_ Muribaculaceae*, which produce short-chain fatty acids (SCFAs) from fermenting dietary polysaccharides, are observed to be decreased (Shaidullov et al., 2021; Yamaguchi et al., 2021), which is consistent with our results. It is widely recognized that SCFAs play a crucial role in regulating inflammation, a process intricately linked to the pathophysiology of CHF. SCFAs are also integral in preserving the integrity of the gut barrier, modulating immune responses, and serving as an energy source for colonocytes. *Ruminococcus* is a genus within the Ruminococcaceae family. Species within the *Ruminococcus* genus are mainly known for designing cellulose and resistant starches, thereby contributing to the fermentation process that produces SCFAs (Michels et al., 2022). These metabolic activities are vital for maintaining a healthy gut environment and the host’s overall metabolic health (Zhu et al., 2019). The bacterial levels listed above could be restored to varied degrees following SFI treatment. In addition, results from a study by LEfse showed that the SCFA producers, *Ruminococcaceae* and *Lachnospiraceae*, were significantly overrepresented in the SFI_M group, these findings reveal that SFI is instrumental to SCFA-producing bacteria.

Gut microbiota can affect an organism by modulating the host metabolic products or through its metabolic products. In a metabolomics study by Li et al. (2023), metabolic imbalances such as disturbances in the metabolism of amino acids, lipids, and glucose, were established to be associated with heart failure (Li et al., 2023). LC-MS analysis was conducted to investigate the influence of gut microbiota on the host metabolism of the ISO-induced HF rat model. In our research involving metabolites, 48 differential metabolites were detected in the HF rat model, and all the metabolites showed a callback trend, with 28 significantly changed metabolites. These findings indicate that the SFI has a regulating effect on the metabolic disturbances induced by HF. Untargeted metabolomics have become a powerful tool for evaluating the efficacy of TCM. As a cutting-edge technological system in biology, metabolomics embraces a holistic perspective akin to TCM, enabling in-depth exploration of TCM’s complex conditions and multifactorial nature. Tian et al. used untargeted metabolomics to investigate the mechanism of action of QFPDD in treating coronavirus-induced pneumonia in mice (Tian et al., 2022). UPLC-Q-TOF/MS is also used for TCM syndrome differentiation (Ye et al., 2021).

The results from our KEGG pathway analysis revealed that the regulation of metabolites by SFI focuses on the pentose phosphate pathway, pyrimidine metabolism, and purine metabolism. Glycometabolism, recognized for its critical function in energy production, encompasses three primary metabolic pathways: oxidative phosphorylation, glycolysis, and the pentose phosphate pathway. It is vital to maintain cardiovascular health to ensure the efficient progression of glycometabolism (Peng et al., 2023). D-ribose is a pentose sugar present in every living cell and is a critical component of several crucial biomolecules such as RNA, nucleotides, and riboflavin. It is synthesized from glucose via the pentose phosphate pathway (PPP) within the cell. Through the non-oxidative phase of the PPP, d-ribose plays an essential role in producing adenosine triphosphate (ATP). As a result, it is utilized as a therapeutic agent to improve cardiac function in patients with HF. Research shows that Supplemental d-ribose reduces the symptoms of HFpEF and increases EF (Pierce et al., 2022). Our study indicates that SFI can increase D ribose and Sedoheptulose levels. Altogether, these data provide the basis for glycometabolism as an essential target of SFI. Growing evidence suggests that the levels of intermediates from purine degradation reflect the energy status of myocardial cells. Under normal conditions, an increase in the heart’s energy demands a need for increased levels of purine nucleotides and their by-products. In contrast, a considerable decrease in total purine production suggests that myocardial cells are conserving energy, hence maintaining the myocardium’s energy balance. In pathological states, such as myocardial ischemia, ATP breaks down into xanthine and accumulates in the tissue. The produced xanthine is converted into uric acid by xanthine oxidase, which generates a significant amount of superoxide anions, causing cellular damage. Under low oxygen environments, the byproducts of the breakdown of adenosine and inosine serve as more efficient energy sources than extracellular glucose, thereby slowing the build-up of nicotinamide adenine dinucleotide (NADH) and offering a degree of protection to the cells (Wang G. et al., 2022). In the present study, hypoxanthine and deoxyinosine levels were significantly increased in the HF rat model, indicating the presence of an energy imbalance in the HF rat model, which is consistent with research findings (Berry and Hare, 2004).

A significant crosstalk exists between the gut microbiota and the host through substrate co-metabolism and metabolic exchange. Therefore, it may be possible to infer host and microbiome co-metabolism through untargeted metabolomics, as Zhao et al. (2022) showed that interactions between fecal metabolomes and gut microbiomes can reveal functional markers in cerebral ischemic stroke (Zhao et al., 2022). Chen employed fecal metabonomics in conjunction with 16S rRNA gene sequencing to examine alterations in gut microbiota in rats exhibiting kidney-yang deficiency syndrome and the impact of the You-gui pill intervention. Tong’s study found that Gushudan could protect rats with kidney-yang deficient disease by modulating the gut-kidney axis (Tong et al., 2022). MetOrigin is a bioinformatics tool designed to pinpoint the specific bacterial species involved in various metabolic reactions and elucidate their roles in these processes (Fan et al., 2023). In our study, metabolite tracing analysis showed that Taurine and hypotaurine metabolism was found to be specific to the microbial community. The biosynthesis of Pyrimidine metabolism, Purine metabolism, beta-alanine metabolism, Naphthalene degradation, Pantothenate, and CoA biosynthesis were identified as co-metabolic pathways between microbes and hosts. The Spearman correlation analysis in our study also showed a significant correlation between differentially expressed metabolites regulated by SFI and the gut microbiota, indicating that SFI could modulate the co-metabolism of ISO-induced HF.

Our research may give some insights into the relationship between gut microbiota and heart failure. However, the validation of this relevant hypothesis based on animal model testing faces several challenges. Specifically, we need to determine whether the improvement in dysbiosis and metabolome abnormality is merely a consequence of heart failure amelioration or if it plays a critical role in heart failure progression. Additionally, distinguishing changes in gut microbiota induced by heart failure from those induced by specific interventions like ISO is essential. To address these challenges, future studies should consider conducting fecal transplantation trials and expanding both sample sizes and clinical data collection. These steps will enhance our understanding and validation of the relationship between gut microbiota and heart failure.

## 5 Conclusion

This study utilized a comprehensive methodology, incorporating various techniques such as echocardiography, protein chip detection, histopathology, 16S rDNA sequencing, and metabolomics, to investigate the effectiveness of SFI in treating heart failure. The comprehensive findings from our study demonstrated significant benefits of SFI treatment in rats with isoproterenol-induced heart failure. SFI effectively modulated inflammatory imbalance, reduced serum and urine TMAO levels, and improved gut microbiota composition. Specifically, SFI substantially increased the abundance of *Bacteroidota* at the phylum level and enriched SCFA-producing bacteria such as *Ruminococcus* and Erysipelotrichaceae at the genus level. Furthermore, SFI influenced the composition of intestinal metabolites, particularly those associated with the pentose phosphate pathway, pyrimidine metabolism, and purine metabolism. Our findings highlight SFI’s ability to address dysbiosis within the gut microbiota-host co-metabolism.

## Data Availability

The raw data for the 16S rRNA sequencing is available through NCBI with the BioProject accession number PRJNA1096125, available at: https://www.ncbi.nlm.nih.gov/bioproject/PRJNA1096125.

## Supplementary material

SUPPLEMENTARY FIGURE S1
**(A)** PCA score plot of the four groups and QC samples. **(B)** The α-diversity of ACE, Chao, Simpson, and Shannon index. Data are presented as the mean ± SD; n = 6.

SUPPLEMENTARY TABLE S1 The information for all the affected pathways.

SUPPLEMENTARY TABLE S2 The related dataset of Sankey network of Bio-ko00240.

SUPPLEMENTARY TABLE S3 The related dataset of Sankey network of Bio-ko00230.

SUPPLEMENTARY TABLE S4 The related dataset of Spearman’s correlation.

## Abbreviations

HF: Heart Failure. LPS: Lipopolysaccharide. LVEF: Left Ventricular Ejection Fraction. LVFS: Left Ventricular Fractional Shortening. NT-proBNP: N-terminal ProB-type Natriuretic Peptide. OTU: Operational Taxonomic Unit. OPLS-DA: Orthogonal Projections to Latent Structures Discriminate Analysis. PCoA: Principal Coordinates Analysis. PCA: Principle Component Analysis. SCFA: Short-Chain Fatty Acids. SFI: Shenfu Injection. VIP: Variable Importance in the Projection. TMAO: Trimethylamine N-oxide. QC:Quality control. MET:Metoprolol.UHPLC-QE-MS: Ultra-High Performance Liquid Chromatography Quadrupole-Orbitrap Mass Spectrometry.UHPLC-OE-MS: Ultra-High Performance Liquid Chromatography-Ion Trap-Orbitrap Mass Spectrometry. TIC:Total Ion Chromatogram.
