# Supplementary figures and images for: Shenfu injection improves isoproterenol-induced heart failure in rats by modulating co-metabolism and regulating the trimethylamine-N-oxide - inflammation axis
Source: Front Pharmacol. 2024 Jun 20;15:1412300. doi: 10.3389/fphar.2024.1412300 (PMC11222397; doi:10.3389/fphar.2024.1412300)

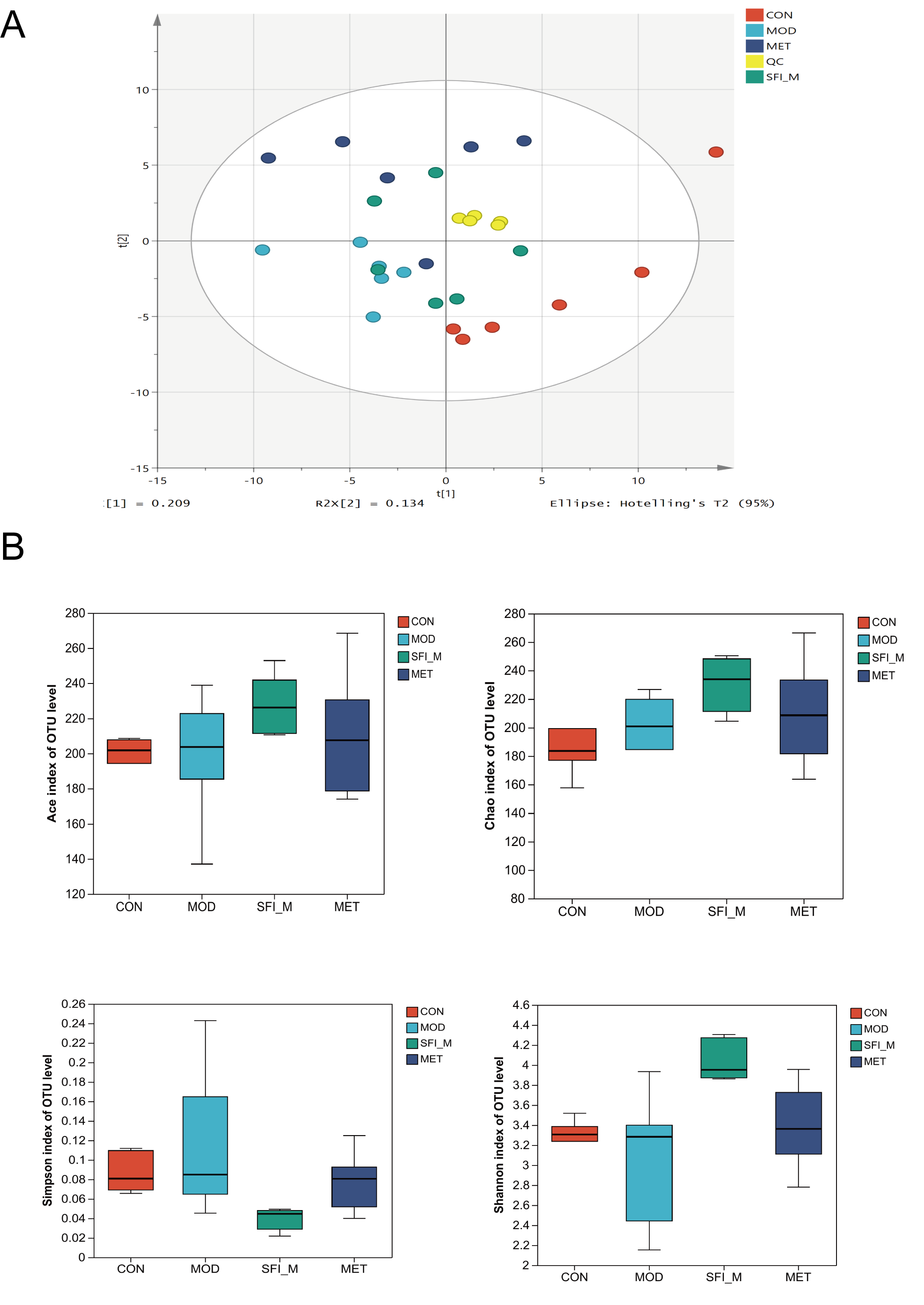

Supplement: Supplementary file 4 [file Image1.TIF]
