# Supplementary material for: Shenfu injection improves isoproterenol-induced heart failure in rats by modulating co-metabolism and regulating the trimethylamine-N-oxide - inflammation axis
Source: Front Pharmacol. 2024 Jun 20;15:1412300. doi: 10.3389/fphar.2024.1412300 (PMC11222397; doi:10.3389/fphar.2024.1412300)
Supplement: Supplementary file 6 [file DataSheet2.DOCX]

1.Metabolites Extraction：

The feces samples (25 mg±1 mg) were taken, mixed with beads and 500 μL of extraction solution (MeOH:ACN:H2O, 2:2:1 (v/v)). The extraction solution contain

deuterated internal standards. The mixed solution were vortexed for 30 s.Then the mixed samples were homogenized (35 Hz,4 min) and sonicated for 5 min in 4 ℃ water bath, the step repeat for three times. The samples were incubated for 1 h at -40 ℃ to precipitate proteins. Then the samples ware centrifuged at 12000 rpm (RCF=13800(×g),R= 8.6cm) for 15 min at 4 ℃. The supernatant was transferred to a fresh glass vial for analysis. The quality control (QC) sample was prepared by mixing an equal aliquot of the supernatant of samples.

2.LC-MS/MS Analysis：

For polar metabolites, LC-MS/MS analyses were performed using an UHPLC system (Vanquish, Thermo Fisher Scientific) with a Waters ACQUITY UPLC BEH Amide (2.1 mm × 50 mm, 1.7 μm) coupled to Orbitrap Exploris 120 mass spectrometer (Orbitrap MS, Thermo).

The mobile phase consisted of 25 mmol/L ammonium acetate and 25 mmol/L ammonia hydroxide in water（pH = 9.75）(A) and acetonitrile (B). The auto-sampler temperature was 4 ℃, and the injection volume was 2 μL. The Orbitrap Exploris 120 mass spectrometer was used for its ability to acquire MS/MS spectra on information-dependent acquisition (IDA) mode in the control of the acquisition software (Xcalibur, Thermo). In this mode, the acquisition software continuously evaluates the full scan MS spectrum. The ESI source conditions were set as following: sheath gas flow rate as 50 Arb, Aux gas flow rate as 15 Arb, capillary temperature 320 ℃, full MS resolution as 60000, MS/MS resolution as 15000, collision energy: SNCE 20/30/40, spray voltage as 3.8 kV (positive) or -3.4 kV (negative), respectively.

3.Data preprocessing and annotation：

The raw data were converted to the mzXML format using ProteoWizard and processed with an in-house program. which was developed using R and based on XCMS, for peak detection, extraction, alignment, and integration. The R package and the BiotreeDB（V3.0） were applied in metabolite identification.

Reference: Zhou, Z., Luo, M., Zhang, H., Yin, Y., Cai, Y., & Zhu, Z. J. (2022).

Metabolite annotation from knowns to unknowns through knowledge-guided multi-layer metabolic networking. Nature communications, 13(1), 6656.
